# Umbelliferone-induced recovery of postsynaptic density structure revealed by the reanalysis of public electron microscopy data

**DOI:** 10.1186/s42649-026-00129-7

**Published:** 2026-06-01

**Authors:** Ga-Young Choi, Hyosung Choi, Seohyeong Lee, Hee-Seok Kweon

**Affiliations:** Center for Bio-imaging & Translational Research, Korea Basic Science Institute, Cheongju, 28119 Republic Of Korea

**Keywords:** Hippocampus, Postsynaptic density, Umbelliferone, Scopolamine, Electron tomography, High voltage electron microscopy, Korea BioData Station (K-BDS)

## Abstract

Public biological data repositories not only ensure research reproducibility and transparency but also enable new scientific discoveries that extend beyond the scope of original studies. A previously published study on umbelliferone (UMB) demonstrated recovery of synaptic vesicle architecture under scopolamine (SCO)-induced pathological conditions, and the corresponding high-resolution electron microscopy dataset was deposited in the Korea BioData Station (K-BDS, https://kbds.re.kr), a national biological data platform.

In this study, we reanalyzed this open dataset to investigate whether UMB also affects the postsynaptic density (PSD), a previously unexplored structural determinant of synaptic efficacy and plasticity. Using the original high-resolution transmission electron microscopy images, we performed secondary quantitative analyses focusing on PSD length, width, and ultrastructural features across experimental groups. Our reanalysis revealed that UMB treatment significantly increased PSD length and width compared with the SCO-treated group, indicating a structural restoration of impaired postsynaptic architecture. Notably, these PSD-level alterations were not described in the original publication, demonstrating that novel biological insights can be derived from existing data through purposeful reuse.

Collectively, this study underscores the value of K-BDS as a national biological data platform that facilitates secondary discovery and advances data-driven neuroscience research without additional animal experimentation.

**Supplementary Information:**

The online version contains supplementary material available at 10.1186/s42649-026-00129-7.

## Description

Umbelliferone (UMB) is a naturally occurring coumarin derivative that has been reported to exert neuroprotective effects in experimental models of memory and cognitive impairment (Hindam et al. 2020; Jiang et al. 2025). In a previously published study, UMB was shown to restore synaptic vesicle organization in the hippocampus under scopolamine (SCO)-induced pathological conditions, based on high-resolution electron microscopy and three-dimensional electron tomography analyses (Choi et al. 2023, 2024). The corresponding ultrastructural datasets were deposited in the Korea BioData Station (K-BDS; https://kbds.re.kr) under accession ID KAP240751 as publicly accessible resources, thereby providing an opportunity for further data-driven investigation beyond the original analysis.

While the original study primarily focused on presynaptic vesicle recovery, the postsynaptic density (PSD) represents an equally critical ultrastructural component of excitatory synapses, serving as a structural and functional hub for synaptic efficacy and plasticity (Kim and Ko 2006; Verpelli et al. 2014). PSD morphology, particularly its length and width, is closely associated with synaptic strength and stability (Hayashi et al. 2009; Meyer et al. 2014). Given the high spatial resolution and preservation quality of the deposited electron microscopy data, we hypothesized that the deposited datasets could support a secondary analysis of PSD structure that was not addressed in the original study. Accordingly, the inclusion of essential contextual metadata was necessary to ensure reproducibility and interoperability. The availability of such metadata within the K-BDS repository enabled robust secondary quantitative and three-dimensional analyses of postsynaptic density architecture.

To test this hypothesis, we reanalyzed publicly available transmission electron microscopy (TEM) and three-dimensional electron tomography datasets derived from hippocampal synapses in four experimental groups: Control, UMB, SCO, and SCO + UMB. Using the original high-resolution images, PSD length and width were quantitatively measured at clearly identifiable synapses, with calibration performed using embedded scale bars and associated metadata to ensure reproducible and accurate measurements across groups. In addition, three-dimensional electron tomography was used to confirm PSD thickness, continuity, and spatial organization, thereby providing complementary structural information to support a more accurate interpretation of ultrastructural changes that cannot be fully captured in single two-dimensional sections.

**Fig. 1:**
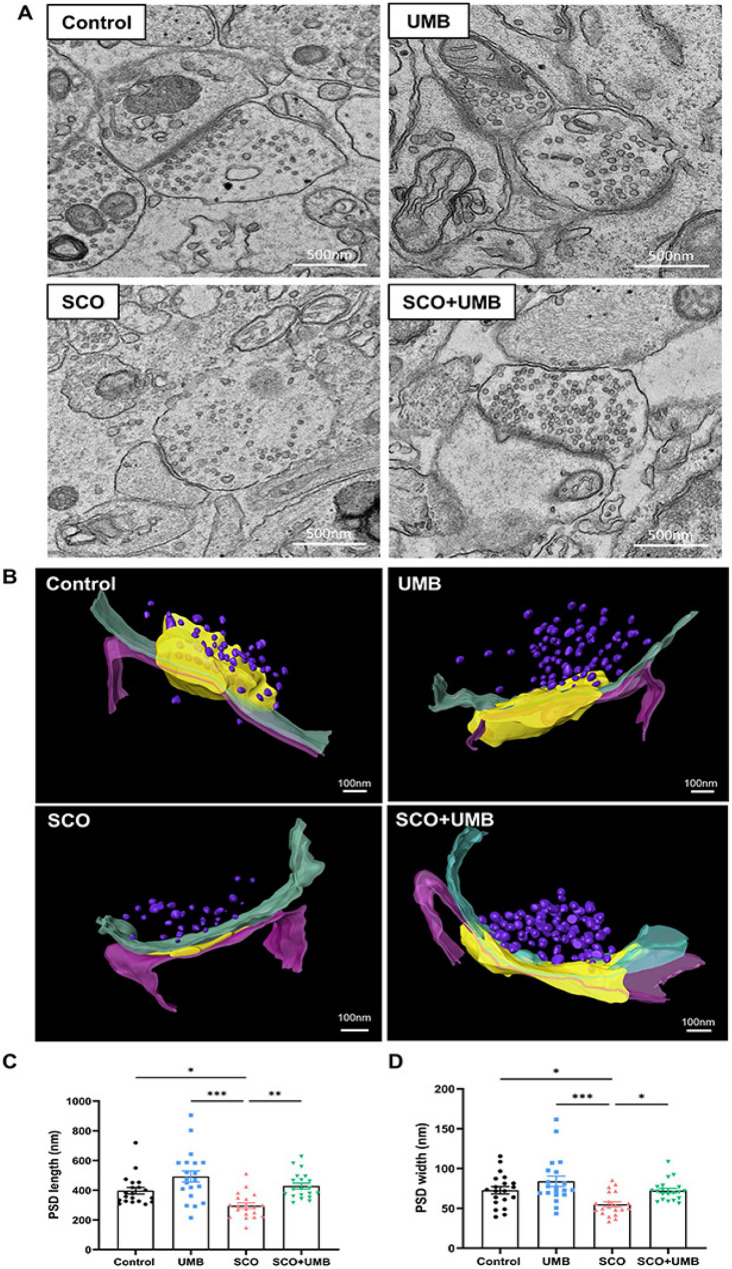
Transmission electron microscopy analysis showing the effects of umbelliferone (UMB) on hippocampal synaptic ultrastructure. **A** Representative TEM images of the postsynaptic density (PSD) in the control, UMB, scopolamine (SCO), and SCO + UMB groups. Scale bar = 500 nm. **B** 3D reconstructions of the PSD (yellow) in each group showing presynapse (green), postsynapse (pink) and synaptic vesicles (purple) of the hippocampal synapses. Scale bar = 100 nm. **C** Average PSD length (nm). **D** Average PSD width (nm). Data were analyzed by one-way ANOVA followed by Tukey’s honest significant difference test. ***p < 0.001, **p < 0.01, *p < 0.05

High-resolution TEM images acquired using the KBSI Bio-HVEM (high voltage electron microscopy) System (JEM-1000BEF; JEOL Ltd., Tokyo, Japan) from the original data were used for quantitative analysis of PSD morphology (Fig. 1A). Images were obtained at magnifications sufficient to clearly resolve postsynaptic densities within the hippocampus. The PSD length was defined as the linear measurement of the electron-dense area along the postsynaptic membrane, taken parallel to the synaptic cleft, whereas the PSD width was measured perpendicularly from the inner side of the postsynaptic membrane outward to the furthest edge of the electron-dense material. The PSD length and width were quantified using ImageJ software (National Institutes of Health, USA), with calibration performed based on the scale bars embedded in the original TEM images. A total of 20 synapses were analyzed from five rats (*N* = 5), with four synapses measured per rat, and these measurements were included in the statistical analysis. Quantitative data are presented as mean ± standard error of the mean (SEM). Statistical analyses were conducted following assessment of data distribution, using one-way analysis of variance (ANOVA) with appropriate post hoc tests for multiple group comparisons. A *p*-value of less than 0.05 was considered statistically significant.

Tomographic reconstruction and three-dimensional visualization were performed using AMIRA software (Thermo Fisher Scientific Inc., Waltham, MA, USA) in Fig. 1B. Reconstructed volumes were examined to assess PSD thickness, continuity, and overall three-dimensional organization, whereas quantitative measurements of length and width were obtained from the two-dimensional TEM images.

Quantitative analysis results revealed differences in PSD length (F(3,76) = 10.0, *p* < 0.001, Fig. 1C) and width (F(3,76) = 6.83, *p* < 0.001, Fig. 1D) among each group. The SCO group (length: 296 ± 18.7 nm, width: 55.1 ± 3.28 nm) showed a significant reduction of PSD length and width compared with the control group (length: 397 ± 22.3 nm, *p* < 0.05; width: 72.8 ± 4.62 nm, *p* < 0.05), indicating marked impairment of postsynaptic architecture under cholinergic dysfunction. In contrast, the UMB only group (length: 493 ± 38.0 nm, *p* = 0.052; width: 84.0 ± 6.54 nm, *p* = 0.312) exhibited a trend toward increased PSD length and width relative to the control, suggesting a potential enhancement of postsynaptic structure. Notably, the SCO + UMB group (length: 429 ± 19.6 nm, *p* < 0.01; width: 72.2 ± 2.87 nm, *p* < 0.05) showed a significant recovery in PSD length and width compared to the SCO group. These findings were consistently supported by three-dimensional tomographic reconstructions, which demonstrated recovery of PSD thickness and continuity in the SCO + UMB group compared with the SCO group.

Importantly, these PSD-related structural changes were not reported in the original study, despite being derived from the same electron microscopy data. This highlights how reanalysis of high-quality public data can uncover additional information that extends beyond the initial research focus. This study demonstrated that UMB-mediated synaptic recovery is not limited to presynaptic vesicles but also involves restoration of postsynaptic density, suggesting coordinated structural remodeling across synaptic compartments and providing a more comprehensive view of synaptic structural plasticity.

Collectively, this study demonstrates that open-access ultrastructural data deposited in national biological data platforms such as K-BDS, when accompanied by well-curated metadata, can be effectively repurposed for secondary quantitative and three-dimensional analyses without the need for additional animal experimentation. This reuse extends the analytical value of the original dataset and supports data-driven investigation in neuroscience research.

## Supplementary Information

Supplementary Material 1.

Supplementary Material 2.

Supplementary Material 3.

Supplementary Material 4.

## Data Availability

Not applicable.
